# Thoracic cord compression due to ligamentum flavum gouty tophus: a case report and literature review

**DOI:** 10.1038/sc.2015.93

**Published:** 2015-06-16

**Authors:** Z-F Zheng, H-L Shi, Y Xing, D Li, J-Y Jia, S Lin

**Affiliations:** 1 Department of Nephrology, General Hospital of Tianjin Medical University, Tianjin, China; 2 Department of Radiology, General Hospital of Tianjin Medical University, Tianjin, China

## Abstract

**Study design:**

Here we describe a patient who developed myelopathy due to gouty tophi of the ligamentum flavum in the thoracic spine. We also review similar cases previously reported in the literature.

**Objective:**

Our aim was to present a case of myelopathy due to thoracic spinal gouty tophus.

**Methods:**

We report the case of a 56-year-old male with history of peripheral gout and renal insufficiency. The patient complained of back pain and paraparesis of the left lower limb. Multiple tophi were noted over several interphalangeal and metatarsophalangeal joints. Neurological examination showed decreased left lower limb strength and a positive Babinski sign. Magnetic resonance imaging of the thoracic spine revealed hypertrophy of the ligamentum flavum at the level of T3/T4, T5/T6, T9/T10, T10/T11 and T11/T12.

**Results:**

A thoracic laminectomy at T1-T5 was performed. Chalky white granular material was found in the ligamentum flavum during surgery. Histological analysis of the specimen demonstrated a gouty tophus. The patient's back pain and paraparesis of the lower left limb improved.

**Conclusion:**

The clinician should include spinal gout in the differential diagnosis when dealing with patients with gout and axial pain with or without neurologic deficits. If this diagnosis is seriously entertained, then a CT scan or magnetic resonance imaging as well as tissue biopsy may be needed to establish the diagnosis.

## Background

Gout is monosodium urate crystal-induced inflammatory arthritis associated with hyperuricemia.^1^ The incidence of gout is estimated to be 0.2–0.4% worldwide, with an annual incidence of 0.01–0.015%.^2^ Gout is more common in men with a male-to-female ratio of 4:1 below the age of 65 years and 3:1 above the age of 65 years.^3^ Predisposing factors for an acute attack include trauma, surgery, increasing alcohol intake, high levels of intake of meat and fish and medications including diuretics and cyclosporine. Tophaceous gout is characterized by precipitation of urate crystals in the joints and periarticular tissues, and deposits commonly are found in the metatarsophalangeal joints, ankles, knees, wrists, fingers and shoulders. Gouty arthritis of the axial joints, particularly of the spine, is very rare.

Gout could involve all the segments of the spine. King *et al.* reviewed the records of reported cases of axial gouty tophi and ~44% of the axial gout patients had involvement of the lumbar vertebrae, 39% the cervical vertebrae and 17% the thoracic vertebrae.^4^ Tophaceous gout could impact anatomic components of the spine, such as facet joint,^5^ vertebral bodies,^6^ pedicle,^7^ lamina^8^ and ligamentum flavum.^9^ Patients present with features of spinal stenosis, lumbar radiculopathy, spondylolisthesis, cauda equine syndrome or spinal infection.

We present a case of a newly diagnosed patient with thoracic spine tophaceous gout involving the ligamentum flavum.

## Case presentation

A 54-year-old Chinese male with a 12-year history of gout and hyperuricemia reported a 3-day history of lower limb edema and elevated serum creatinine. He suffered episodic gouty attacks, despite intermittently being treated with nonsteroidal anti-inflammatory drugs. Tophaceous deposits were present in the hands and toes for at least 5 years. Furthermore, the patient reported high alcohol intake spanning 20 years. He denied any history of trauma or spinal injuries. After he had been admitted to inpatient ward, he was found to have tenderness in the left anterior leg with anesthesia. Five days later, he complained of progressive back pain radiating to his anterior chest. The level of skin anesthesia increased from the lower extremities to his chest. Several days later, the paraparesis progressed to difficulty walking.

On physical examination the patient was found to have a fever of 38.8 °C. He had significant tenderness in his back and obvious difficulty with ambulation secondary to pain. Multiple tophi were also noted over several interphalangeal joints and metatarsophalangeal joints. Neurological examination showed that his lower limb strength had decreased (Grade 4/5) on the left and he had left ankle clonus. The lower abdominal and cremasteric reflexes were normal. The left lower limb also showed exaggerated reflexes as well as positive Babinski and Rossolimo' sign. Reflexes were intact and within normal range on the straight-leg raise test. Laboratory values at the time of admission are depicted in Table 1.

**Figure 1:**
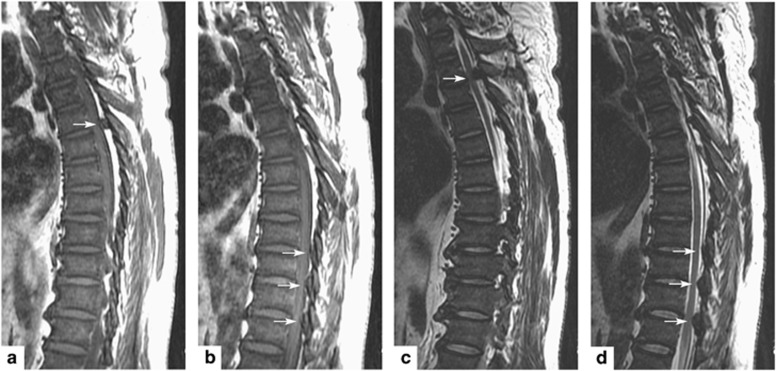
Sagittal image of hypointensity magnetic resonance imaging shows hypertrophy ligamentum flavum. (**a**) T1-weighted image shows the lesion at the T3-T4 level. (**b**) T1-weighted image shows the lesions at the T9-T10, T10-T11 and T11-T12 levels. (**c**) T2-weighted image shows the lesion at the T3-T4 level. (**d**) T2-weighted image shows the lesions at the T9-T10, T10-T11 and T11-T12 levels.

**Figure 2:**
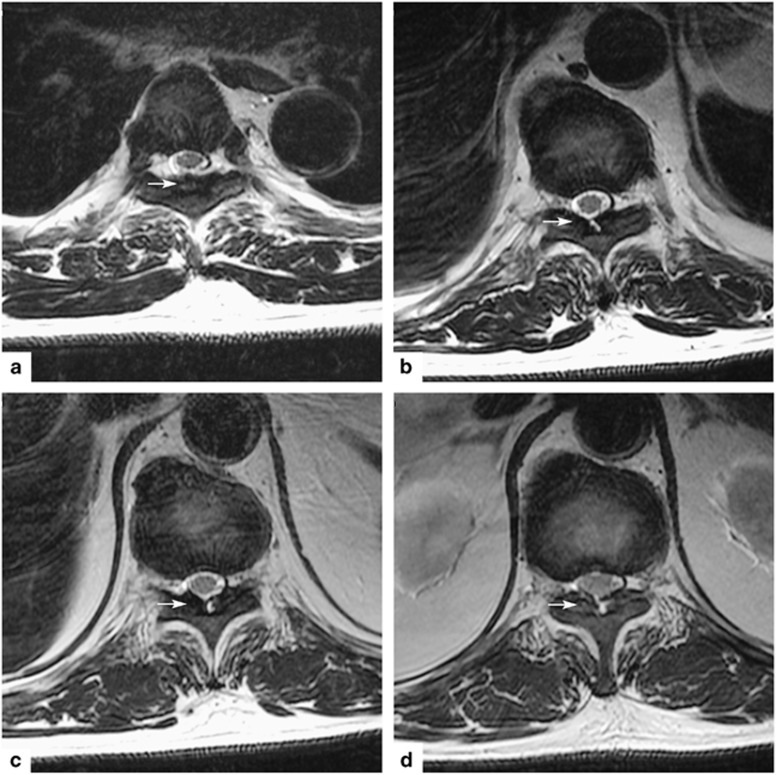
Axial images of hypointensity magnetic resonance imaging shows hypertrophy of the ligamentum flavum on T2-weighted image. (**a**) The lesion at the T3-T4 level. (**b**) The lesion at the T9-T10 level. (**c**) The lesion at the T10-T11 level. (**d**) The lesion at the T11-T12 level.

Computed tomography of the thoracic spine showed spinal stenosis at the T3/T4, T9-T12 levels. He also underwent magnetic resonance imaging, which showed hypertrophic ligamentum flavum at the level of T3/T4, T5/T6, T9/T10, T10/T11 and T11/T12 (Figures 1 and 2). The discs of T7/T8, L3/L4, and L5/S1 showed posterior bulge. Degenerative disc disease was found at the levels of T1/T2, T12/L1, L1/L2 and L5/S1.

A thoracic laminectomy was performed at T1-T5. During the operation, an abnormal mass with a white, chalky, cheese-like and granular appearance was observed. A culture of the chalky material revealed no bacterial growth. A histological examination of the material removed during the laminectomy showed amorphous eosinophilic material with thin needle-shaped crystals that were negatively birefringent on polarizing microscopy. Bacteriologic examinations were negative.

After his operation, his back pain and skin anesthesia of the lower extremities was markedly improved. He was prescribed allopurinol and transferred to a rehabilitation facility.

## Discussion

**Figure 3:**
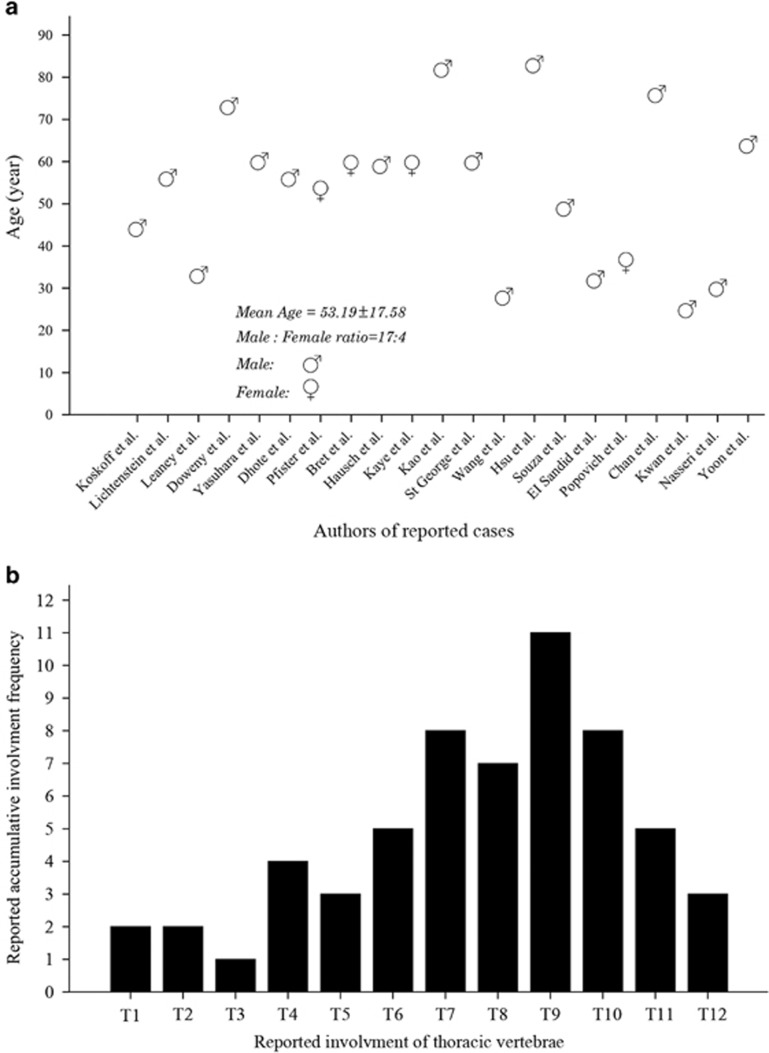
Distribution characteristics of reported tophaceous gout of the thoracic spine in the literature. (**a**) Distribution of the patient's age. (**b**) Distribution of the involved location.

The first radiologic and pathologic description of gouty involvement of the spine was published by Kersley *et al.* in 1950.^10^ However, the first case of thoracic gouty spine patient was not discussed until the report by Koskoff *et al.* in 1953.^11^ To the best of our knowledge, 21 thoracic spine cases have been reported. The reported cases of spinal gout involvement of thoracic vertebrae are listed in Table 2.^7, 11, 12, 13, 14, 15, 16, 17, 18, 19, 20, 21, 22, 23, 24, 25, 26, 27, 28, 29, 30^ There was significant gender difference with a male-to-female ratio of 17:4 in the reported cases. Fourteen (66.7%) patients reported a history of gout symptom ranging from 2 to 35 years.^11, 12, 13, 15, 16, 17, 18, 19, 20, 22, 23, 24, 25, 30^ Peripheral tophi were found in ten (47.6%) patients.^7, 11, 12, 13, 15, 16, 17, 18, 19, 25^ Although tophi were reported in all thoracic regions, the most frequent involvement was seen in the thoracic region at T7-T10 (Figure 3). The most common location of gouty tophi involvement was extradural space.^11, 13, 14, 15, 16, 18, 21, 22, 25, 26^ Other locations of axial elements, such as facet joints,^23, 29, 30^ discs,^12^ vertebral bodies,^7, 12, 17, 19, 20^ pedicles^7, 13, 27, 30^ and costovertebral joint^28^ have been reported. However, the formation of gouty tophi within the thoracic spine involving the ligamentum flavum causing spinal cord compression, as occurs in our case, have only been reported previously by Wang *et al.*^23^ and Hus *et al.*^24^

The prevalence of spinal gout is unclear since most of the available information comes from anecdotal case-reports. Konatalapalli *et al.*^31^ reviewed 630 patients who were diagnosed with gouty arthritis, tophaceous gout or unspecified gout. Sixty-four patients had computed tomography images of cervical, thoracic or pelvic region. Spinal gout was identified in 9 of these 64 patients (14%). More recently, Konatalapalli *et al* accomplished a cross-sectional study regarding axial gout. Seventeen of the 48 subjects (35%) had computed tomography evidence of spinal gout and 7 (15%) had spinal tophi.^32^ On the basis of these observational studies, we speculated that the prevalence of axial gouty tophi was grossly underestimated.

Although the etiopathogenesis of the crystal accumulation in the axial skeleton is not completely known, it has been reported that factors such as degenerative disease of the spine, necrosis of the tissues or previous injuries can trigger the process.^4, 33, 34^ Meanwhile, some predisposing factors such as old age, low temperature, low serum pH level, renal insufficiency, diuretic and cyclospine A agent, IgA nephropathy and high alcohol intake are thought to promote tophi formation and development. The reason for the involvement of peripheral joints in gout is considered to be related to the decrease of the solubility of the crystals in the places with lower temperature and formation of tophi in avascular tissues.^4, 35^ In addition, lower blood pH causes a decrease in the binding plasma proteins and trauma causes an increase in the precipitation of urate crystals, both of which cause an increase in tophus formation.^4, 35, 36^ In our review, most of the patients with gouty tophi involved T7 through T10, which was consistent with the possibility that inflammation associated with motion-related damage may create an environment favorable for urate deposition. Renal dysfunction plays a significant role in raising the uric acid levels of the serum. Primary or secondary renal function promotes uric acid levels, causing tophi deposition in the spine and mild spinal stenosis with abnormal nerve compression. In turn, the elevated serum acid level further impairs renal function and contributes to the worsening of tophi deposition, which is supported by Chonchol *et al.*^37^ Our patient had a long history of hyperurecimia, with only intermittent pharmaceutical control. Subcutaneous deposition of gouty tophi in the right elbow area was also noted at this time. Thus, a relatively low environmental temperature and decreased renal urate clearance may be prerequisites for urate deposition.

Clinical manifestations of thoracic spinal gout range from back pain, unilateral or bilateral extremities paralysis, limbs weakness, sensory impaired to urinary retention. Neurologic symptoms were dependent on the level of the spine that was affected.

On MR, spinal tophi appear as homogeneous areas of intermediate-to-low signal intensity on T1-weighted images. On T2-weighted images, the signal intensity of the tophi varies from homogeneous hyperintensity to homogeneous hypointensity. This hyperintensity may result from a relative increase in the water content of the tophus and the relative homogeneity of local magnetic field within the tophi. In comparison, the T2-weighted hypointensity may be caused by immobile protons in the tophi. This appearance can be due to regions of calcifications, mature fibrous tissue, or hemosiderin deposition in the tophi. After gadolinium enhancement, the tophi show homogeneous or heterogeneous marginal enhancement. The enhancement of the tophi is thought to be the result of well-vascularized chronic, inflammatory fibrous tissue engendered by urate crystal deposition.^4, 24^

Gouty tophi are nodular, chalky white in the center, made of monosodium urate crystals, proteins, and mucopolysaccharides. Under microscopy, the urate depositions are found to be surrounded by multinucleated histiocytes, which are giant cells with foreign bodies associated with lymphoplasmocytic cells and fibroblasts. Moreover, monosodium urate crystals can be dissolved by formalin. This may be a reason why in our case there were no birefringent crystals under polarized light. It is important that the specimen should be properly fixed after biopsy or operation.

Surgical decompression such as laminectomy followed by optimization of pharmacological treatment can improve the patient's clinical symptoms and provide a good prognosis. Modification of risk factors such as alcohol consumption, improvement in renal function, or alteration of the diuretic regimen may be beneficial and should be pursued whenever possible. Frequent follow-ups and imaging studies may permit early diagnosis and minimized complications of this disease.

## Conclusion

In conclusion, although spinal gout maybe rare, it is important to be aware of this possibility. The clinician should include spinal gout as a differential diagnosis when dealing with patients with gout and axial pain with or without neurologic deficits. Even a short, uncontrolled period of time in the course of the disease could lead to devastating neurologic deficits necessitating emergent surgery for decompression. If this diagnosis is seriously entertained, then a computed tomography scan or magnetic resonance imaging as well as tissue biopsy may be needed to establish the diagnosis. If gout is suspected at the time of the biopsy, this needs to be communicated to the pathologist because monosodium urate crystals will dissolve during routine histologic processing.

## Figures and Tables

**Table 1:** Laboratory data at presentation

*Variable*	*Result*	*Reference range*
*Blood count*		
Leukocytes (10^9 ^l^−1^)	9.05	4.0–10.0
Erythrocytes (10^12^ l^−1^)	**3.12**	4.0–5.5
Hemoglobin (g dl^−1^)	**90**	120–160
Hematocrit (%)	**26.4**	42.0–49.0
Platelet count (10^9^ l^−1^)	247	100–300

*Serum chemistry*		
Total protein (g l^−1^)	66	62–85
Albumin (g l^−1^)	40	35–55
Globulin (g l^−1^)	26	26–37
Total cholesterol (mmol l^−1^)	4.66	3.59–5.17
Triglyceride (mmol l^−1^)	**2.1**	0.57–1.71
Low density lipoprotein (mmol l^−1^)	2.52	1.33–3.30
Lactate dehydrogenase (U l^−1^)	245	94–250
Glutamic pyruvic transaminase (U l^−1^)	16	5–40
Glutamic oxaloacetic transaminase (U l^−1^)	13	8–40
Blood urea nitrogen (mmol l^−1^)	7.6	1.7–8.3
Serum creatinine (μmol l^−1^)	**120**	44–115
Serum uric acid (μmol l^−1^)	320	140–414
Fasting blood glucose (mmol l^−1^)	**6.2**	3.6–5.8

*Immunology*		
Immunoglobulin G (mg dl^−1^)	991	751–1560
Immunoglobulin A (mg dl^−1^)	171	82–453
Immunoglobulin M (mg dl^−1^)	47	46–304
Immunoglobulin E (IU ml^−1^)	116	<165
Complement C3 (mg dl^−1^)	152	79-152
Complement C4 (mg dl^−1^)	38	16-38
C-reactive protein (mg dl^−1^)	**12.9**	<0.8
Circulating immune complexes (U ml^−1^)	3.2	<13
Antinuclear antibodies	Negative	Negative
Antibody against double-stranded DNA	Negative	Negative
Antibodies to extractable nuclear antigens	Negative	Negative
Anti-neutrophil cytoplasmic antibodies	Negative	Negative
Anti-glomerular basement membranous antibody (RU ml^−1^)	<20	<20
Anti-cardiolipin antibody	Negative	Negative
Anti-ribosomal P-protein antibody	Negative	Negative
Serum immunofixation electrophoresis	Negative	Negative
Anti-cyclic citndlinated peptide antibody (U ml^−1^)	6.4	<12.0
Anti-mutated citrullinated vimentin antibody (U ml^−1^)	5.2	<20.0
Anti-RA33 antibody (U ml^−1^)	11.3	<25.0
Anti-keratin antibody	Negative	Negative
Anti-perinuclear factor antibody	Negative	Negative
Rheumatoid factor IgA subclass (U ml^−1^)	8.8	<12.0
Rheumatoid factor IgG subclass (U ml^−1^)	10.3	<12.0

The bold and italic entries indicate abnormal values beyond the reference range.

**Table 2:** Demographic and clinical characteristics of patients with tophaceous gout of the spine

*Author*	*Publication year*	*Country*	*Age (years)*	*Gender*	*History of gout symptom (years)*	*Neurological symptoms*	*Clinical description of gout*	*Serum uric acid level (μmol l^−1^)*	*Level of involvement*	*Location of involvement*	*Means of diagnosis*	*Treatment*	*Evaluation*
Koskoff *et al.*	1953	United States	44	M	12	Bilateral legs paralysis; back aching pain; bilateral lower extremities weakness	Severe, polyarticular, tophi	625	T9-T11	Extradural space	Operation	Decompression	Improved
Levin *et al.*	1956	United States	56	M	35	Normal	Severe, polyarticular, tophi	774	T12-L1	Disc; vertebral bodies	Autopsy	ND	NA
Leaney *et al.*	1983	Australia	33	M	5	Midthoracic pain; bilateral lower limbs paralysis and weakness; urinary retention	Severe, polyarticular, tophi	560	T7-T11	Extradural space; pedicles	Operation	Laminectomy	Improved
Downey *et al.*	1987	United Kingdom	73	M	NR	Bilateral legs paralysis; gait disturbance	NR	NR	T1	Extradural space	Operation	NR	NR
Yasuhara *et al.*	1994	Japan	60	M	5	Back pain; hypesthesia; bilateral lower extremites weakness	Mild, Polyarticular tophi	619	T6-T7	Extradural space	Operation	Laminectomy	Improved
Dhote *et al.*	1997	France	56	M	2	Bilateral lower extremites paralysis and weakness	Severe, polyarticular, tophi	929	T4-T9	Extradural space	Operation	Laminectomy	Improved
Pfister *et al.*	1998	United State	53	F	25	Back pain;urinary retention; unilaterial right leg weakness	Severe, polyarticular, tophi	NR	T8-T9	Vertebral bodies	Needle biopsy	Laminectomy	Improved
Bret *et al.*	1999	France	59	F	16	Bilateral lower extremities paralysis and weakness	Severe, polyarticular, tophi	340	T2-T9	Extradural space	Operation	Laminectomy	Improved
Hausch *et al.*	1999	United State	59	M	3	Back pain;	Polyarticular, tophi	726	T4;T7	Vertebral bodies	Needle biopsy	Conservative	Improved
Kaye *et al.*	1999	South Africa	59	F	4	Bowel and bladder dysfunction; bilateral lower extremites weakness; back pain	Polyarticular, no tophi	NA	T8	Vertebral bodies	Operation	Laminectomy	NR
Kao *et al.*	2000	Taiwan	82	M	5	Bilateral lower extremites weakness; urinary retention	Polyarticular, no tophi	506	T10-T11	Extradural space	Operation	Laminectomy	Improved
St George *et al.*	2001	United Kingdom	60	M	10	Unilateral left leg weakness	No tophi	NR	T1-T2	Extradural space	Operation	Laminectomy	Improved
Wang *et al.*	2001	Taiwan	28	M	5	Back pain; bilateral lower extremities paralysis; urinary retention	Polyarticular, no tophi	601	T9-T10	Facet joint; ligamentum flavum	Operation	Laminectomy	Resovled
Hsu *et al.*	2002	Taiwan	83	M	2	Bilateral lower extremites weakness and numbness	No tophi	375	T9-T11	Ligamentum flavum	Operation	Laminectomy	NR
Souza *et al.*	2002	Brazil	49	M	5	Back pain; unilateral right leg weakness; sensory impairment	Severe, polyarticular, tophi	NR	T9-T10	Extradural space	Operation	Laminectomy	Resovled
EI Sandid *et al.*	2004	United States	32	M	NA	Back pain	No tophi	620	T7-T9	Extradural space	Operation	Laminectomy	Improved
Popovich *et al.*	2006	United States	36	F	NA	Bilateral lower extremites weakness; sensory impaired	No tophi	571	T4-T7	Pedicles	Operation	Laminectomy	Resovled
Chan *et al.*	2009	Hong Kong	76	M	NA	Bilateral lower extremites weakness	Severe, polyarticular, tophi	NR	T8;T10	Pedicles; vertebral bodies	Needle biopsy	Conservative	Improved
Kwan *et al.*	2013	Canada	25	M	NR	Back pain	No tophi	462	T9;T10;T12	Costovertebral joint	Needle biopsy	Conservative	Decreased
Nasseri *et al.*	2013	United States	30	M	NR	Back pain; bilateral lower extremities weakness	NR	NR	T10-T11	Facet joint	NR	NR	NR
Yoon *et al.*	2013	Korea	64	M	8	Back pain; bilateral lower extremities weakness	NR	726	T6-T7	Facet joint; pedicles	Operation	Laminectomy	Improved

Abbreviations: F, female; M, male; NR, not report; ND, not done; NA, not applicable.
